# Comparison of Dexmedetomidine Versus Fentanyl-Based Anesthetic Protocols Under Patient State Index Guidance in Patients Undergoing Elective Neurosurgical Procedures with Intraoperative Neurophysiological Monitoring

**DOI:** 10.7759/cureus.35864

**Published:** 2023-03-07

**Authors:** Jerry Jame Joy, Prasanna U Bidkar, Srinivasan Swaminathan, Mukilan Balasubramanian, Ankita Dey, Vivek Chandar Chinnarasan, Adethen Gunasekaran

**Affiliations:** 1 Department of Anesthesiology and Critical Care, Jawaharlal Institute of Postgraduate Medical Education and Research, Puducherry, IND; 2 Department of Anesthesiology, Cleveland Clinic, Abu Dhabi, ARE; 3 Department of Anesthesiology, All India Institute of Medical Sciences, Pondicherry, IND

**Keywords:** intraoperative neurophysiological monitoring (ionm), total intravenous anesthesia (tiva), neurosurgery., patient state index, dexmedetomidine, propofol

## Abstract

Objectives

The study was designed to elucidate the effects of dexmedetomidine as an anesthetic adjunct to propofol in total intravenous anesthesia (TIVA) on anesthetic dose reduction, the quality of intraoperative neurophysiological monitoring (IONM) recordings, analgesic requirements, and recovery parameters in patients undergoing neurosurgical procedures with neurophysiological monitoring.

Methods

A total of 54 patients for elective neurosurgical procedures with IONM were randomized to group D (dexmedetomidine) and group F (fentanyl). A loading dose of the study drug of 1µg/kg followed by 0.5 µg/kg/h infusion was used in two groups. Propofol-based TIVA with a Schneider target-controlled infusion model was used for induction and maintenance with effect site concentration of 4-5 and 2.5-4 µg/mL, respectively, titrated to a Patient State Index (PSI) of 25-40. Baseline IONM recordings were obtained after induction. The mean propofol consumption, number of patient movements, quality of IONM recordings, number of fentanyl boluses, hemodynamic characteristics, and recovery parameters were recorded.

Results

The mean propofol consumption was significantly lower in group D when compared to group F (101.4 ± 13.5 µg/kg/min vs 148.0 ± 29.8 µg/kg/min). Baseline IONM recordings were acquired in all patients without any difficulty. The two groups were comparable with respect to the number of additional boluses of fentanyl, patient movements, and recovery characteristics.

Conclusion

Dexmedetomidine as an adjuvant to propofol in TIVA reduces the requirement of the latter, without affecting the IONM recordings. The addition of dexmedetomidine also ensures stable hemodynamics and decreases the requirement of opioids with similar recovery characteristics.

## Introduction

Intraoperative neurophysiological monitoring (IONM), used for assessing the functional integrity of neural structures in anesthetized patients, is now an irrevocable part of neurosurgical procedures [1,2]. The primary objective of IONM is to ensure safe surgery by detecting early neurological insults and preventing irreversible damage to neural structures [1,3]. The use of motor evoked potentials (MEPs) and somato-sensory evoked potentials (SSEPs) as part of IONM is the current gold standard practice [4], but because the commonly used anesthetic agents interfere with their intraoperative readings, the drug regimen for such surgeries must be tailored to facilitate faultless neuromonitoring. This can be accomplished by using a propofol-opioid-based total intravenous anesthesia (TIVA) regimen, as advocated by the American Society of Neurophysiological Monitoring [5,6].

However, high concentrations or prolonged use of propofol-based TIVA are known to cause a reduction in the MEP amplitude and fade, and can lead to prolonged awakening, lipemia, platelet dysfunction, and, rarely, propofol infusion syndrome [7,8]. To prevent these adverse sequelae and to curtail propofol consumption without affecting evoked potentials, the modified Delphi consensus recommends adding an adjuvant such as ketamine, dexmedetomidine, or lidocaine to a standard TIVA regimen [9,10].

Dexmedetomidine is a sought-after choice in anesthetic practice as an adjuvant for its sympatholytic, sedative, anesthetic-sparing, and hemodynamic stabilizing properties without causing significant respiratory depression. The pharmacodynamic interaction between dexmedetomidine and propofol has been shown to decrease the requirement for propofol [11-15], but its effect on evoked potentials remains unclear [16-20]. Dexmedetomidine as an adjuvant has also been shown in studies to reduce opioid requirements due to its α-2 agonist property [21-27].

This study was designed to compare the consumption of propofol between two TIVA regimes, using either dexmedetomidine or fentanyl along with propofol, under patient state index (PSI) guidance, in patients undergoing neurosurgical procedures with IONM. We also compared the quality of IONM recordings, the number of patient movements in the intraoperative period, the number of additional boluses of fentanyl required, and hemodynamic parameters between the two groups.

## Materials and methods

We conducted a prospective, randomized controlled trial comparing dexmedetomidine and fentanyl-based anesthetic protocols under PSI guidance in patients undergoing elective neurosurgical procedures with IONM.

This trial was approved by the local Institutional Ethics Committee (JIP/IEC/2020/018) and registered on Clinical Trials Registry of India (CTRI/2020/07/026896). Written informed consent was obtained from all participants or next of kin. Patients aged 18 to 60 years and belonging to the American Society of Anesthesiologists (ASA) grades 1 and 2 scheduled for elective cranial and spinal neurosurgical procedures with IONM were prospectively enrolled in the trial. Exclusion criteria were patients with a history of allergy to any of the study medications, patients with heart rate less than 60 per minute, pregnant or nursing mothers, patients with cardiac arrhythmias, patients with hepatic, renal, or cardiac dysfunction, and surgical factors that hindered the placement of PSI sensor electrodes.

Randomization

**Figure 1:**
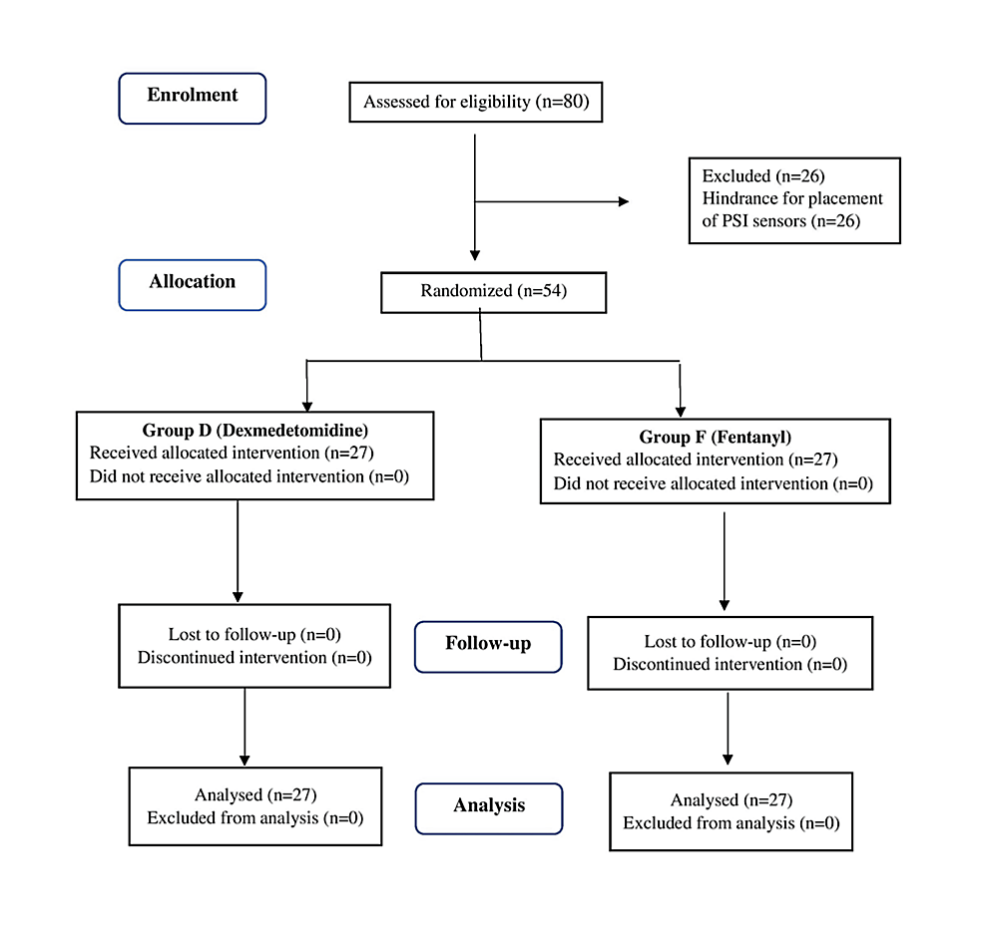
CONSORT diagram of the study

Patients were randomized to receive either dexmedetomidine (group D) or fentanyl (group F) by a computer-generated double-blinded stratified randomization system (www.randomiser.org) (Figure 1). Concealment was ensured using the Serially Numbered Opaque Sealed Envelope (SNOSE) method. A single researcher, who was not involved in data collection or patient follow-up, opened the envelope. The study drugs (dexmedetomidine or fentanyl) were administered to the patients according to the group allocation. The anesthesiologist conducting the study and the patients were blinded to the study drugs.

Anesthesia

Intraoperative monitoring consisted of non-invasive blood pressure (NIBP) and invasive blood pressure, electrocardiography (ECG), and pulse oximetry (SpO2). PSI obtained from the SEDLine™ (Masimo Corporation, Irvine, CA, USA) monitor was used to monitor the depth of anesthesia. The six electrode sensors were applied in accordance with the manufacturer's instructions after the forehead skin was cleansed with ether to increase skin conductivity. Patients were instructed to close their eyes for two minutes after the sensors were applied, and the baseline PSI values and corresponding spectral edge frequency 95 (SEF95) values were recorded. Group F received fentanyl and group D received dexmedetomidine as follows: intravenous loading dose of 1 µg/kg over 10 minutes followed by an intraoperative maintenance infusion of 0.5 µg/kg/hr. After the loading dose of the study drug, anesthesia was induced with fentanyl 1µg/kg and propofol using a target-controlled infusion (TCI) pump with a target effect site concentration of 4-5 µg/mL, in accordance with the Schneider model [26] in both groups. Rocuronium 1 mg/kg was administered to facilitate endotracheal intubation. Following intubation, anesthesia was maintained with propofol with target effect-site concentrations ranging from 2.5 to 4 µg/mL in both groups. The depth of anesthesia was titrated to maintain PSI between 25 and 50, and ventilation was adjusted to maintain end-tidal carbon dioxide between 32-36 mm Hg with fractional inspired oxygen concentration of 0.4 (air in oxygen). To aid in intraoperative IONM, further doses of rocuronium were avoided. Hemodynamic parameters such as heart rate, mean arterial blood pressure (MAP), PSI, and SEF95 were noted at baseline and every 15 minutes till the end of surgery. A bolus of fentanyl 0.5 µg/kg was administered if the PSI was within the target range but the heart rate and systolic blood pressure (SBP) had increased by more than 20% from baseline. The number of additional boluses of fentanyl given was noted. Any episode of intraoperative patient movements was recorded, and the propofol effect site concentration was increased by 0.5 µg/ml till the episode ceased. Movements that took place during MEP recordings were excluded.

At the commencement of skin closure, study drug infusions were stopped and propofol infusion was tapered with proper control of hemodynamics. Patients who fulfilled the clinical criteria for extubation were extubated at the end of surgery. Various recovery characteristics such as emergence time, extubation time, and recovery time were noted. Those patients who could not be extubated were transferred to the neurosurgical intensive care unit for ventilatory support and further management.

IONM recordings

Standard half-inch subcutaneous needles were used to set up SSEPs and MEPs at all stimulating and recording sites. All the IONM was conducted by the same neurophysiologist in both groups. Standard locations on the ankle and wrist were used to stimulate the tibial and median nerve SSEPs, respectively. Active electrodes were used to record SSEPs at the lateral scalp for the median nerve (C3' and C4' are 2 cm posterior to C3 and C4 sites, respectively) and the midline scalp (Cz') for the tibial nerve. Anodal pulses were administered by electrodes positioned at C3 and C4 for MEP stimulation. A reference electrode was placed over the tendon of the muscle, and an active electrode was inserted over the belly of the contralateral thenar and abductor hallucis muscles to record MEPs. For stimulation and recording, a NIM-ECLIPSE® E4 Nerve Monitoring System by Medtronic (Minneapolis, MN) was employed. Filter settings for SSEPs and MEPs ranged from 30 to 1,000 Hz. Analysis time was 50 milliseconds for median nerve stimulation and 100 milliseconds for tibial stimulation. A pulse of a 0.2-millisecond duration and an intensity of 50 and 25 milliamperes was used to activate the tibial and median nerves. A train of 4 to 8 pulses with an interstimulus period of 2 milliseconds and an intensity ranging from 150 to 400 volts was used to stimulate MEPs.

Baseline SSEP and MEP recordings were recorded and measured whenever necessary. Any 10% increase in latency or a 50% drop in amplitude from baseline readings of SSEP, and any 50% reduction in amplitude of MEP recordings from any of the target muscle groups were recorded.

Data collection

Demographic (age, sex, weight, ASA grade) and intraoperative (heart rate, SBP, diastolic blood pressure, and mean arterial pressure, duration and type of surgery, patient movement, emergence time, extubation time, recovery time, and patients requiring mechanical ventilation) data were recorded.

The total amount of propofol used was computed at the end of the procedure, and the mean propofol consumption was determined as µg/kg/min by dividing the total amount of propofol used intraoperatively by the duration of infusion and body weight. The total fentanyl dose and the number of extra boluses of fentanyl administered intraoperatively for both groups were noted.

Statistical analysis and sample size calculation

The estimated sample size was based on the anticipated variations in propofol maintenance dose between the two groups. As per our previous case records, the mean propofol consumption was 150 ± 25 μg/kg/min. Considering a mean reduction of propofol of 15% with 90% power and 0.05 level of significance, the sample size was calculated to be 27 in each group.

All statistical analyses were carried out using SPSS Version 19.0 (IBM Corp., Armonk, NY). A frequency and percentage scale was used to express the distribution of categorical characteristics, and continuous variables were expressed as mean ± standard deviation. The comparison of the main outcome variable was done using an independent student t-test. The categorical variables were compared between the groups using the chi-square test. All statistical analyses were carried out at a significance level of α error of 5%.

## Results

A total of eighty patients scheduled for neurosurgical procedures requiring IONM were screened for eligibility; 26 were excluded due to the hindrance of placement of PSI sensors and the remaining 54 were included in the study (Fig.1). The two groups were comparable in terms of gender, ASA category, duration of surgery, and type of surgery (Table 1).

**Table 1:** Patient characteristics ASA, American Society of Anesthesiologists

Parameter		Group D (Dexmedetomidine) (n=27)	Group F (Fentanyl) (n=27)	P-Value
Gender	Male	16 (59.3%)	16 (59.3%)	1
	Female	11 (40.7%)	11 (40.7%)	
ASA category	I	16 (59.3%)	12 (44.4%)	0.276
	II	11 (40.7%)	15 (55.6%)	
Age (year)		36.9 ± 9.5	43.1 ± 11	0.030
Weight (kg)		62.9 ± 9.7	56.9 ± 9.3	0.025
Duration of surgery (minutes)		354.4 ± 125.1	362.2 ± 124	0.819
Type of surgery	Intracranial	8 (29.6%)	12 (44.4%)	0.26
	Spinal	19 (70.4%)	15 (55.6%)	

**Figure 2:**
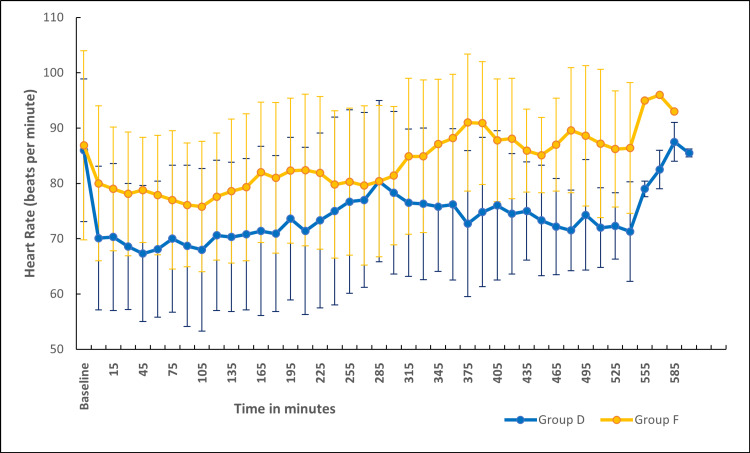
Comparison of heart rate between the groups The mean heart rate was significantly lower in group D in comparison to group F except at a few time points viz. 225, 240, 255, 270, 285, 300, 315, 330, 495, 510, 575, and 585 minutes.

**Figure 3:**
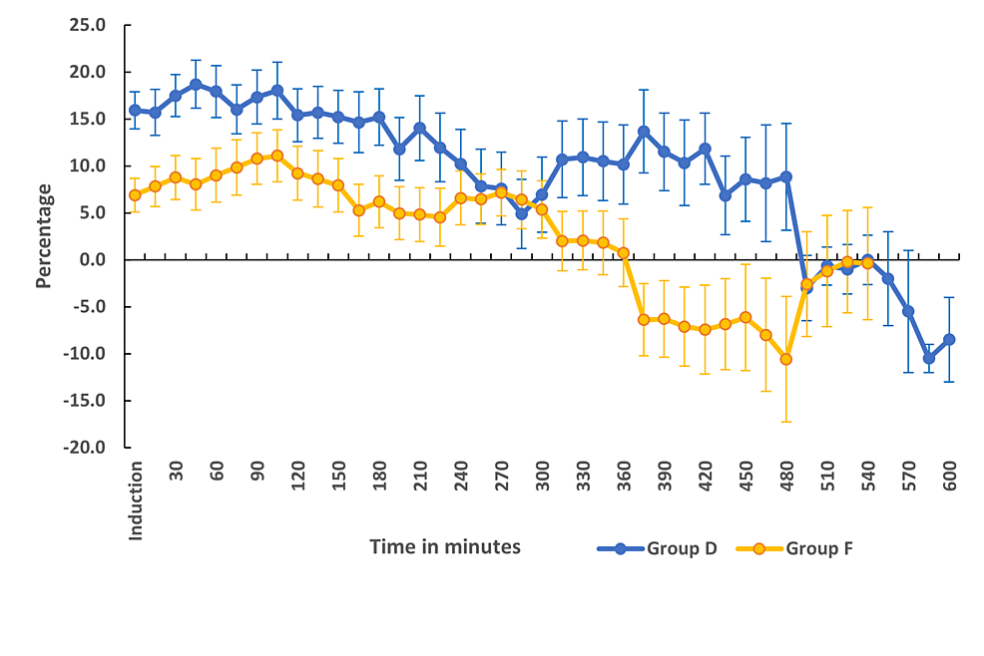
Comparison of percentage change in heart rate from the baseline in two groups A positive value indicates a decrease from the baseline and a negative value indicates an increase from the baseline

**Figure 4:**
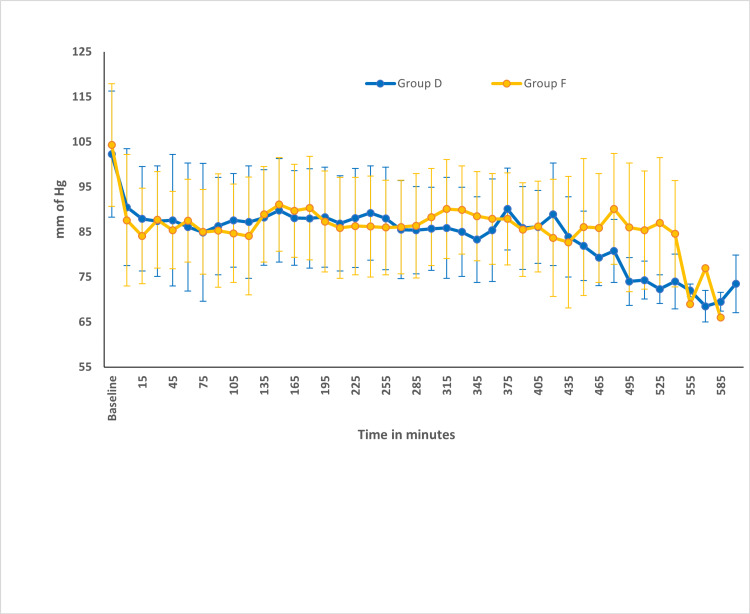
Comparison of MAP between the groups MAP, mean arterial blood pressure

**Figure 5:**
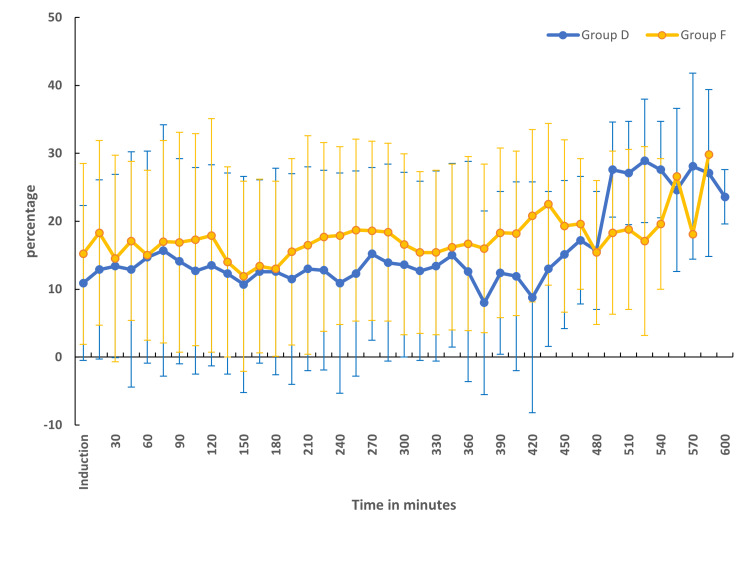
Comparison of percentage change in MAP from the baseline in two groups MAP, mean arterial blood pressure

The baseline heart rates were comparable between the two groups. The mean heart rate was significantly lower in group D in comparison to group F (Figure 2). The percentage change in heart rate from baseline was statistically significant and higher in group D when compared to group F (Figure 3). There were no episodes of bradycardia in either of the groups (Figure 2). MAP was comparable between both groups at baseline and at various time points (Figure 4). The percentage change in MAP was also comparable between the groups (Figure 5).

**Figure 6:**
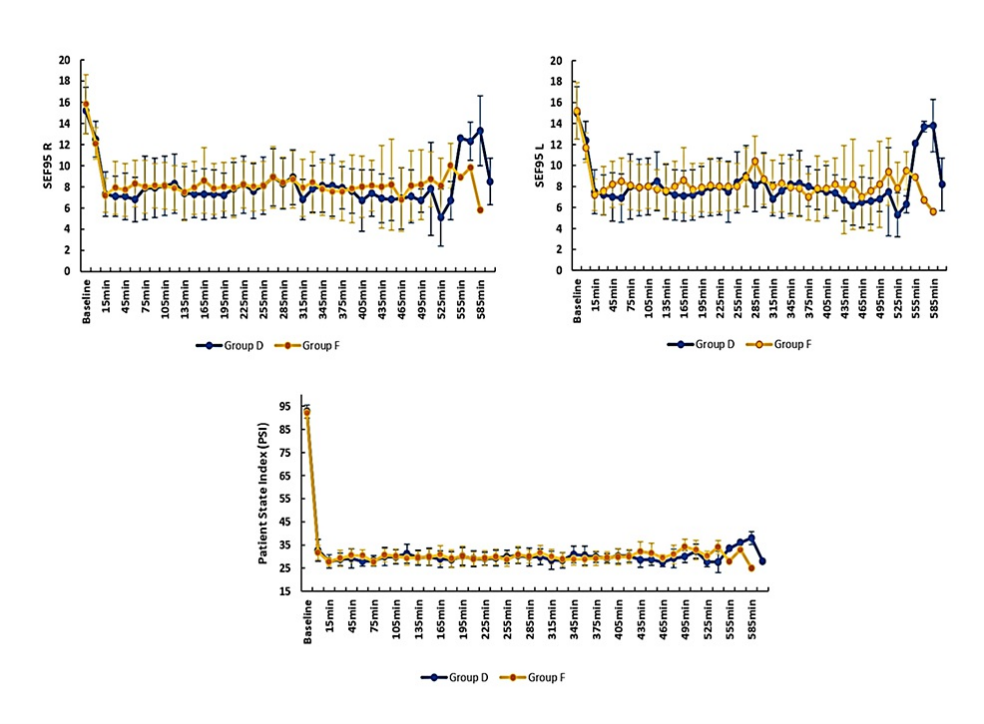
Comparison of PSI, SEF95 right, and SEF95 left between the groups PSI, patient state index; SEF95, spectral edge frequency 95

**Figure 7:**
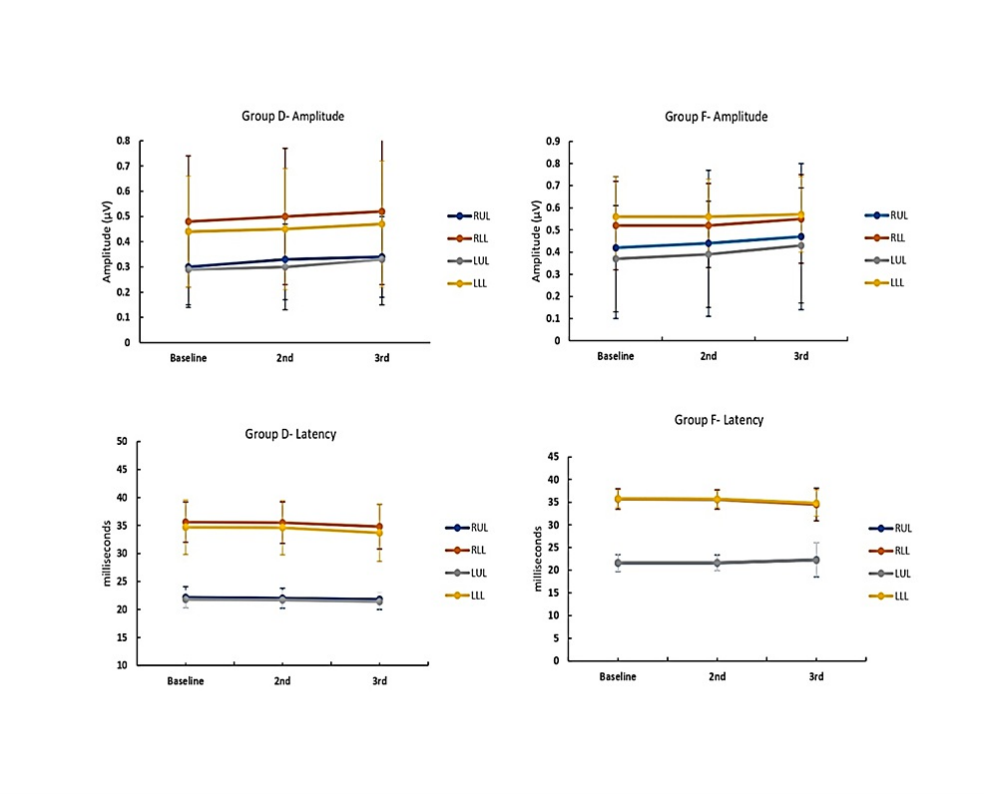
Comparison of SSEPs between two groups SSEP, somato-sensory evoked potential

**Figure 8:**
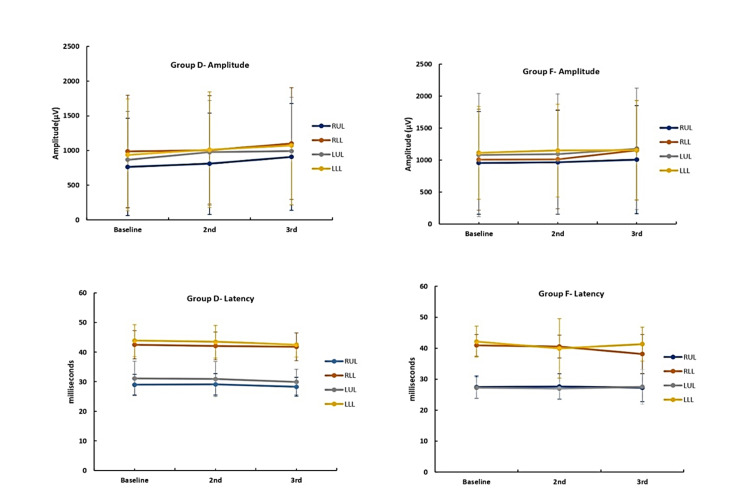
Comparison of MEPs between two groups MEP, motor evoked potential

The mean propofol consumption in group D (101.4 ± 13.5 µg/kg/min) was lower when compared to group F (148.0 ± 29.8 µg/kg/min), with a statistically significant difference (p<0.05) (Table 2). The number of additional fentanyl boluses and the total intraoperative fentanyl requirement were comparable between the two groups (group D 42.6 ± 26.9 vs group F 50.2 ± 21.0) (Table 2). The two groups did not have a statistically significant difference in terms of the number of intraoperative patient movements recorded and were comparable with respect to PSI and SEF95 measured on the left side and the right side (Figure 6). Overall, 19 patients in group D and 11 patients in group F were extubated on table, and their recovery characteristics (emergence time of 9.3 ± 2.0 vs 8.7 ± 1.6, extubation time of 11.7 ± 2.5 vs 10.5 ± 1.7, recovery time of 13.6 ± 2.9 vs 12.3 ± 2.1) were also comparable(Table 3). The SSEP and MEP were recordable in all patients in both groups and were comparable with their baseline in terms of amplitude and latency (Figures 7, 8).

**Table 2:** Intraoperative propofol consumption, fentanyl requirements, and patient movements between the groups

Drug	Group D (Dexmedetomidine) (n=27)	Group F (Fentanyl) (n=27)	P-Value
	Mean ± SD	Mean ± SD	
Total propofol (mg)	2290.7 ± 985.4	3052.3 ± 1295.1	0.019
Mean propofol (µg/kg/min)	101.4 ± 13.5	148.0 ± 29.8	< 0.001
Total additional fentanyl (µg)	42.6 ± 26.9	50.2 ± 21.0	0.329
Number of additional fentanyl boluses			
0	12 (44.4%)	5 (18.5%)	0.148
1	8 (29.6%)	9 (33.3%)	
2	6 (22.2%)	9 (33.3%)	
3	1 (3.7%)	4 (14.8%)	
Number of times the patient moved in the intraoperative period			
1	9 (33.3%)	8 (29.6%)	0.073
2	2 (7.4%)	6 (22.2%)	
3	0 (0.0%)	4(14.8%)	
5	1 (3.7%)	0 (0.0%)	

**Table 3:** Recovery characteristics

Parameters	Group D (n=19) Mean ± SD	Group F (n=11) Mean ± SD	P-Value
Emergence time (min)	9.3 ± 2.0	8.7 ± 1.6	0.404
Extubation time (min)	11.7 ± 2.5	10.5 ± 1.7	0.157
Recovery time (min)	13.6 ± 2.9	12.3 ± 2.1	0.182
Mechanical ventilation (n)	8	16	

## Discussion

This randomized controlled trial was designed to compare dexmedetomidine versus fentanyl-based anesthetic protocols under PSI guidance in patients undergoing elective neurosurgical procedures with IONM. The primary outcome of our study was comparing the mean propofol consumption between the two groups intraoperatively. We postulated that the total amount of propofol consumed will be significantly lower in the dexmedetomidine-based anesthetic protocol when compared to the fentanyl-based protocol.

Mean propofol consumption

The mean propofol consumption was lower in the dexmedetomidine group (101.4 ± 13.5 µg/kg/min) when compared to the fentanyl group (148 ± 29.8 µg/kg/min) by approximately 30% and was statistically significant (p < 0.001) (Table 2). Our finding was at par with other studies, demonstrating a propofol-sparing effect of dexmedetomidine due to its α-2 agonist activity on the locus ceruleus [12-15,19,28,29,30-39]. Chakrabarti et al. [33] reported similar findings in patients undergoing cerebellopontine angle surgeries with TIVA when dexmedetomidine was used as an adjuvant. Andleeb et al. [16] compared various adjuvants (ketamine, dexmedetomidine, normal saline) in patients requiring TIVA with IONM and found a significant reduction in propofol consumption in the dexmedetomidine group.

Quality of IONM recordings

In our study, the IONM recording quality was assessed with respect to the baseline, which was obtained successfully in both groups. The target medications had no impact on signal acquisition during the surgery. There was no instance where the dosage of the study drug needed to be modified in order to record the evoked potentials. The usage of dexmedetomidine as an adjuvant to TIVA and its impact on evoked potentials has received little attention from research. In the study by Tobias et al. [34], the administration of dexmedetomidine 1 µg/kg loading dose to the first patient resulted in a decrease in MEP amplitude along with a decrease in bispectral index (BIS) values. Hence the propofol infusion was adjusted to maintain the desired levels which resulted in the reappearance of normal MEP waveforms. Using the protocol thus formulated, dexmedetomidine had no further impact on recording the baseline SSEPs and MEPs. Similarly, Rozet et al. [31] obtained reliable and consistent MEP recordings at 0.6 µg/kg/h dexmedetomidine infusion. Dexmedetomidine was added to the propofol-remifentanil target-controlled infusion regime by Li et al. [40] to study the effect on MEPs. They found no significant changes in amplitude and latency in intergroup and intragroup analysis. Andleeb et al. [16] demonstrated that the baseline median amplitude was comparable in three groups receiving dexmedetomidine, ketamine, and saline. At 60 minutes and at the end of the surgery, there was an increase in the amplitude of MEP in the ketamine group and dexmedetomidine group (significantly higher in the ketamine group) with no changes in the latencies. Lee et al. [36] in their study observed that the baseline amplitude was lower in the dexmedetomidine group and required higher stimulation currents for achieving the baseline values. Similar requirements of higher stimulation intensities were also observed in the study by Pacreu et al. [35] in cranial surgeries without significantly affecting the amplitude and latency of SSEPs and MEPs. These did not hinder the correct interpretation of the responses throughout the surgery. In our study, the neurophysiologist did not report any difficulty nor required higher stimulation currents for obtaining evoked potential recordings both during baseline and intraoperatively in either group.

Additional fentanyl boluses

The requirement for additional intraoperative fentanyl boluses was comparable between the two groups in our study (Table 2). The majority of the studies on TIVA employ a propofol-opioid combination as the standard regimen [16,35]. Our study differs from those studies, as the opioid (fentanyl) is not a part of the standard TIVA regime in either of the groups. The replacement of fentanyl with dexmedetomidine in group D did not increase the analgesic requirements among patients belonging to the group. In fact, the number of additional fentanyl boluses administered was higher in the fentanyl group (23 in group D vs 39 in group F), though the difference was not statistically insignificant (Table 2). This reinforces the analgesic efficacy of dexmedetomidine and its capability to be used as a sole drug in TIVA along with propofol, thus circumventing the opioid-related side effects.

Similar results were obtained by Chakrabarti et al. [33], where the total fentanyl consumption and the additional fentanyl boluses were significantly lower in the dexmedetomidine group. Fentanyl was used in both groups, and this is probably why the additional fentanyl requirement was lower in the dexmedetomidine group in their study. Andleeb et al. [16] reported that fentanyl consumption in dexmedetomidine and ketamine groups was lower than in the saline group, though the difference was not statistically significant. In their respective studies, Wu et al. [28] and Ngwenyama et al. [23] found conflicting results to our study. They found that the addition of dexmedetomidine to the TIVA regimen did not reduce the intraoperative opioid needs. In both investigations, the major analgesic used was remifentanil, which, unfortunately, cannot be compared to fentanyl in our study.

Number of patient movements

The intense surgical stimulus during operations may produce patient movements under anesthesia without neuromuscular blockade. In our study, the number of patient movements was comparable between the two groups (Table 2). No complications were observed due to intraoperative patient movements in our study. The movements of the patient during surgery while under TIVA without neuromuscular blockade have not been compared in any other study so far, to the best of our knowledge.

Inadequate depth may be the predominant cause of patient movements while under anesthesia. However, in our study, PSI was used throughout the surgery in order to keep the depth of anesthesia within the target range of 25-40. Sebel et al. [40] showed that the choice of anesthetic agents affects the predictive value of BIS for movement to skin incision. Movement is correlated with BIS when medications such as propofol or isoflurane are primarily used. However, the correlation is diminished with the addition of adjuncts such as opioids. Katoh et al. [41] in their study observed that none of the electroencephalogram indicators, including BIS, SEF95, and median power frequency, was a reliable guide to the adequacy of anesthesia in preventing movement to incision when using sevoflurane. Some observers hypothesized that the movements of anesthetized patients are mediated subcortically and that cortical function monitors may not be the best way to predict responsiveness to noxious stimuli [42].

Hemodynamics

Dexmedetomidine, being an α-2 agonist, inhibits the central sympathetic outflow, resulting in a reduction in heart rate and occasionally hypotension [43,44]. In our study, the mean heart rate was significantly lower in the dexmedetomidine group in comparison to the fentanyl group (Figure 2). Up until about the fourth hour of anesthesia, the heart rate disparity persisted. This was most likely the consequence of administering a bolus dose of dexmedetomidine followed by an infusion. In the study conducted by Andleeb et al. [16], the heart rate was lower in the dexmedetomidine group compared to the ketamine and saline groups. But this difference became noticeable only after an hour of infusion as a dexmedetomidine bolus dose was not given. In a small subgroup of patients undergoing prolonged surgery, the significance was noted again after 7 hours (Figure 2). However, none of the patients in either group experienced an episode of bradycardia that warranted the administration of atropine. The percentage change in MAP was within 20% from the baseline in both groups (Figure 5). The MAP was comparable between the two groups at all points in time (Figure 4).

Spectral edge frequency 95

The two groups were comparable with regard to the SEF95 left side and right side at all time points. To the best of our knowledge, SEF95 in the context of IONM has not been studied so far.

Recovery characteristics

In our study, the two groups were comparable with respect to their recovery characteristics (Table 3). The sedative effect of dexmedetomidine, which likely nullifies the effect of the lower dose of propofol, can be used to explain the similar recovery characteristics in both groups in our investigation, despite the lower dose of propofol used in the dexmedetomidine group. Similar findings were also observed in the study conducted by Chakrabarti et al. [33]. These results from their study, however, cannot be applied to ours because the study population, anesthetic technique, and surgeries were different.

In a study by Turgut et al. [29], a longer extubation time was seen in the dexmedetomidine group, yielding contradictory results to our study. However, since they compared dexmedetomidine and remifentanil as the primary analgesics, this finding is probably intuitive. Siddiqui et al. [30] also observed longer extubation times in the dexmedetomidine group when compared to the fentanyl group. This difference may be due to the dissimilarity in anesthetic technique and the shorter duration of the procedure, as the study population comprised of patients undergoing laparoscopic cholecystectomies.

A few studies have found a dexmedetomidine-induced reduction in the extubation time that is statistically significant [13,15] In their study on supratentorial tumors, Soliman et al. found a shorter extubation time with dexmedetomidine compared to the saline group [13]. However, because sevoflurane with a muscle relaxant was employed in the studies, the results cannot be extended to our investigation. In a study by Gopalakrishna et al. [15] in patients following transsphenoidal pituitary operations, the extubation time was shorter in the dexmedetomidine group compared to the saline group. This difference may be explained by the different anesthetic methods and the shorter length of the procedure in comparison to our study.

Limitations of the study

Our study is not without its limitations. Firstly, there was no comparison of the propofol induction doses across the two groups. We did not measure the plasma concentration of propofol, dexmedetomidine, or fentanyl. Also, the administration of fentanyl boluses was left at the discretion of the residing anesthesiologist. An Analgesia Nociception Index (ANI) monitor guided therapy, and assessment of the post-operative sedation scores and analgesic requirements in these patients would have added more objectivity to our study. Also, we did not study the cost-effectiveness of the drugs used in the present study.

## Conclusions

We conclude that the use of dexmedetomidine as an adjuvant to propofol in TIVA in patients undergoing surgeries with IONM reduces the requirement of propofol with adequate depth of anesthesia. The use of dexmedetomidine did not interfere with IONM recordings. The addition of dexmedetomidine also provides stable hemodynamics as well as decreases the requirement of opioids with similar recovery characteristics. Though, not significant, there was a trend toward lesser patient movements during intraoperative period.
